# Divisive Faultlines and Knowledge Search in Technological Innovation Network: An Empirical Study of Global Biopharmaceutical Firms

**DOI:** 10.3390/ijerph18115614

**Published:** 2021-05-24

**Authors:** Long Cheng, Meng Wang, Xuming Lou, Zifeng Chen, Yang Yang

**Affiliations:** School of Economics and Management, Xi’an University of Posts & Telecommunications, Xi’an 710061, China; chenglong@xupt.edu.cn (L.C.); wangmeng@stu.xupt.edu.cn (M.W.); chenzifeng0722@126.com (Z.C.); yang.yeah0322@163.com (Y.Y.)

**Keywords:** health, biopharmaceutical, technological innovation network, divisive faultlines, knowledge search, structural holes

## Abstract

Divisive faultlines caused by the uneven distribution of relationship strength play an essential role in knowledge search in the technological innovation network, which serves as an important requirement for the technological innovation network’s macro level to expand to the meso-subgroup level and promote its healthy development. Given that the biopharmaceutical industry, as a high-tech industry, plays a vital role in promoting healthy development, this paper uses the joint patent applications of global biopharmaceutical firms from 2003 to 2018 as a sample to construct a technological innovation network, to explore the relationship between divisive faultlines and knowledge search in the technological innovation network. We also study the moderating effect of structural holes in this relationship. The empirical results show that divisive faultlines significantly affect the depth of knowledge search in the technological innovation network. Divisive faultlines have an inverted U-shaped effect on the breadth of knowledge search in the technological innovation network. Structural holes positively moderate the relationship between divisive faultlines and depth of knowledge search but negatively moderate the inverted U-shaped relationship between divisive faultlines and breadth of knowledge search. This research reveals the relationship between divisive faultlines and the knowledge search in the technological innovation network. The research results provide a theoretical basis and management enlightenment to improve biopharmaceutical firms’ knowledge search ability and promote healthy and sustainable development.

## 1. Introduction

In recent years, with the frequent occurrence of global public health events, health issues have become the focus of global attention. Biopharmaceuticals use modern bioengineering technology to create drugs with special curative effects, which play an essential role in treating significant diseases that seriously threaten human health [1,2]. Many countries in the world unanimously cultivate the biopharmaceutical industry as a new economic growth point, accelerating to seize the commanding heights of “biological economy” [3]. China regards biopharmaceuticals as one of the strategic emerging industries, an essential support for Healthy China. Lalor et al. [4] confirmed that biopharmaceuticals are closely related to developing the national economy and improving people’s healthy life quality. A statistical report by the US market research company IMS Health in 2017 pointed out that the global biopharmaceutical industry’s market size is developing rapidly. Its sales growth rate exceeds the growth rate of the gross world product (GWP).

With the accelerating economic globalization process and the increasingly complex market environment, the biopharmaceutical industry’s sustainable development is inseparable from rich knowledge, technology, and other resources [5]. As an effective way for firms to acquire diversified knowledge, a knowledge search helps enterprises enrich their knowledge base, improving their competitive advantages [6]. As an emerging technology industry, the biopharmaceutical industry usually faces problems such as high risk, high investments, and long returns on investments in the research and development of biopharmaceuticals [7]. It is difficult for biopharmaceutical firms to rely on their limited resources and ability to acquire diversified knowledge to ensure the successful research and development of new drugs. Therefore, firms will shift from closed innovation to open innovation and achieve success in technological innovation activities through cooperative innovation with other innovation subjects [8]. The technological innovation network composed of multiple firms or organizations through cooperative innovation relationships is an important carrier and organizational form for cooperative innovation activities between enterprises [9,10]. Su and Vanhaverbeke [11] found that technological innovation networks can improve corporate knowledge search efficiency and have a significant effect on integrating innovation resources and enhancing innovation capabilities. Balachandran et al. [12] confirmed that firms could acquire heterogeneous knowledge and resources in the innovation network to improve innovation performances. In practice, the United States has formed industrial clusters with the five major biotechnology industrial areas of San Francisco, Boston, Washington, North Carolina, and San Diego to improve the country’s pharmaceutical technology capabilities and promote the development of the biopharmaceutical industry.

Research has proven that the technological innovation network is conducive to the firms’ knowledge search, such as in the early drug discovery phase [13]. However, with the diversification of subjects and inter-firm relationships in the technology innovation network, firms have a preference in choosing innovation partners. Firms tend to maintain strong relationships with partners who have a historical basis for cooperation or high trust, while maintaining weak relationships with other partners, resulting in the uneven distribution of the strength of the relationships between firms in the technological innovation network [14]. The research further finds that, with the diversification of subjects and inter-firm relationships in the technology innovation network, the uneven distribution of the inter-firm relationship strength will lead to obvious agglomeration or alliances among members in the network, and this small group formed by the close connections among members is defined as a subgroup [15]. Subgroups are a common phenomenon in technological innovation networks. Jojo et al. [16] built a technological innovation network based on the alliance of 203 biopharmaceutical firms around the world and found that there are a large number of subgroups in the network, and cohesive subgroups will affect the knowledge search among firms. Heidl et al. [17] proceeded from the perspective of the modular structure at the meso level, such as alliances or factions and found that the uneven distribution of the strength of the relationship between firms will trigger the group discontinuity mechanism, which makes the technological innovation network divide into multiple subgroups and affects the knowledge flow and knowledge search in the technological innovation network. Yi et al. [18], from the perspective of similarity selection and interactions between enterprises, found that divisive faultlines have a negative impact on knowledge sharing in the technological innovation network.

Although existing studies have proven that there are divisive faultlines in the technological innovation network and impact on the knowledge search and performance of firms, few empirical studies use a knowledge search as a dependent variable at the meso subgroup level. Whether and how divisive faultlines affect knowledge searches in technological innovation networks has not been thoroughly elucidated to date. This paper introduces the theory of divisive faultlines from the level of the meso subgroup to explore the mechanism of the impact of divisive faultlines on a knowledge search in the technological innovation network. In addition, because firms are embedded in technological innovation networks, the opportunities for knowledge exposure to firms in different network locations are various. In particular, firms occupying structural holes have information advantages and control advantages in the innovation network, which will affect the knowledge search of enterprises to a large extent [19,20]. Therefore, it is necessary to explore the moderating effect of structural holes on the relationship between divisive faultlines and knowledge search. By using a sample of global biopharmaceutical firms, this research incorporates divisive faultlines, knowledge searches, and structural holes into the same framework, which is helpful to understand the impact of divisive faultlines on a knowledge search in the technological innovation network, and the moderating effects of structural holes on them, and to provide a theoretical basis and a new perspective for improving the efficiency of a knowledge search in biopharmaceutical firms.

The contributions of this paper are mainly reflected in the following aspects: First, the existing knowledge search research primarily focuses on the micro self-centered network level and the macro integrated network level. However, there are few studies from the meso subgroup level. This paper investigates the impact of divisive faultlines on a knowledge search in the technological innovation network, which enriches the literature in the research field of the influencing factors of the knowledge search. Second, based on the existing research, this paper introduces divisive faultlines to the network level. Combined with the characteristics of the technology innovation network, we analyze divisive faultline connotations from the perspective of relationship embeddedness and study the manifestation of divisive faultlines in the innovation network. It enriches the related research on divisive faultlines and makes up for the lack of attention paid to divisive faultlines at the firm and network levels. Finally, based on the structural holes theory, we explore the structural hole moderating effect on the relationship between divisive faultlines and a knowledge search. As structural factors, structural holes can affect the role of factors at the level of relationships between members, which provides a new perspective for firms on how to use the positive effect of divisive faultlines and avoid the negative effect of divisive faultlines to improve the ability of a knowledge search and promote sustainable development.

The paper is organized as follows. Section 2 introduces the literature review. Section 3 proposes the hypotheses and theoretical models. Section 4 shows the research design. Section 5 shows our empirical results. Conclusions are given in the last section.

## 2. Literature Review

### 2.1. Relevant Research on Technological Innovation Network and Knowledge Search

To solve the standing problems of uncertainty, resource scarcity, and limited innovative ability inside the firm in a modern innovation environment, the technology innovation network, a form of network organization based on common innovation goals is established by collaborators between firms and others or organizations [10,21]. The concept of an innovation network was first proposed by Freeman [22], who believed that an innovation network is the basis for systemic innovation, and a network is mainly constructed by innovation cooperation between firms. Jianbo et al. [23] found that firms’ research and development (R&D) cooperation in the technological innovation network can overcome innovation impediments, share R&D costs, gather resources, exchange technology, and share benefits. For example, Shanghai Pudong Zhangjiang Hi-Tech Park’s Pharmaceutical Research Institute once cooperated with the Green Valley Holding (Group) in the park on drug research and development. The Green Valley Holding (Group) provided funds, and the Pharmaceutical Research Institute invested in technological achievements; the cooperation greatly improved the R&D progress and completed phase I, II, and III clinical trials in just over one year.

A knowledge search refers to the specific search strategy that firms choose to realize innovation. Petruzzelli et al. [24] pointed out in the research that the technological development of the biopharmaceutical industry needs to rely on the search and integration of a large amount of diversified knowledge for a long time. At present, domestic and foreign scholars have studied the connotation of a knowledge search from different perspectives, such as open innovation theory, transaction cost theory, and social capital theory, and the dimensions of a knowledge search are divided from a variety of different perspectives [25,26]. According to different search strategies, the existing research divides a knowledge search into two dimensions: depth of the knowledge search and breadth of the knowledge search. Since these two dimensions can more accurately reflect the scope and degree of a knowledge search, they are adopted by more and more scholars [27]. Therefore, this paper follows this division method and divides the knowledge search into two dimensions: depth of the knowledge search and breadth of the knowledge search. Among them, the depth of knowledge search refers to the frequency of repeated visits and existing knowledge by firms, emphasizing the focus of knowledge [28]. The breadth of knowledge search refers to the extent of the fields and channels involved in a firm’s search for external knowledge, emphasizing the diversification of knowledge [29].

The related research of the technological innovation network believes that [30] the close connection between firms encourages members to excavate and revisit existing knowledge continuously and deepen the depth of the knowledge search. Simultaneously, the sparse connections among firms provide channels for firms to search for external knowledge, which is conducive to firms acquiring diversified knowledge [31]. However, some scholars believe that [32] too close or too sparse relationships between firms will limit firms’ opportunities to acquire novel knowledge and hinder firms’ innovation performances. This study believes that the uneven distribution of the strength of inter-firm relationships will affect the depth and breadth of a knowledge search, and the mechanism of uneven distribution of the strength of interorganizational relationships on the depth and breadth of a knowledge search is not clear. Therefore, it is necessary to clarify the influence mechanism of the uneven distribution of the strength of inter-firm relationships on the depth and breadth of a knowledge search.

### 2.2. Related Research on Divisive Faultlines

Divisive faultlines refer to the internal differentiation tendency of the whole network caused by the difference in the degree of shared experience among members in the process of firms’ interactive innovations, which is an important factor in forming and developing subgroups in the technological innovation network [33]. Take China’s COVID-19 vaccine development as an example; in order to maximize the success rate and speed of COVID-19 vaccine research and development, the scientific research team divided the development of a COVID-19 vaccine into five main technical routes: live vaccines, adenovirus vector vaccines, attenuated influenza virus vector vaccines, recombinant protein vaccines, and nucleic acid vaccines, which will also mean that the research and development of the new crown vaccine is mainly divided into five subgroups. There are differences in the shared experiences between members of the various subgroups; members in the same subgroup will form close cooperation, and the relationships between different subgroups will be sparse, so that the technological innovation network formed with the goal of COVID-19 vaccine research and development will cause potential divisive faultlines.

In the literature, related scholars have conducted research on divisive faultlines. The concept of divisive faultlines was first proposed by Lau and Murnighan [34] in the study of team diversity. Those team members with multiple identical attributes (e.g., gender, race, age, and other demographic attributes) may have strong cohesion, so that the team is divided into two or more different subgroups, and the members of each subgroup have high similarities. On this basis, Heidl et al. [17] believed that there are also divisive faultlines in the technological innovation network, because the partner selection tendency of “establishing strong relationships based on historical cooperation” will cause the cohesion of firms on a local scale, resulting in experience between partners. The degree of sharing is different, resulting in the challenges of subgroups of “within the group” and “outside the group”. With deepening research, Zhang et al. [35] and Zhang and Guler [36] found that divisive faultlines have a significant influence on the formation of cooperation between firms and the changes of network members. Xinghua et al. [33] and Long et al., [37] extended divisive faultlines between firms to the technological innovation network, believing that the pre-relationship and the embeddedness of various relationships would cause divisive faultlines, making the members of the subgroup more willing to establish cooperative relations with familiar firms and more inclined to search for knowledge within the known range. This study believes that the uneven distribution of the strength of relationships between firms will cause potential divisive faultlines, making the technological innovation network present the characteristics of local close connections and sparse global connections. Firms will choose different knowledge search strategies in the process of innovation. Therefore, it is necessary to explore divisive faultlines’ influential mechanisms on the depth and breadth of a knowledge search from the meso subgroup level.

### 2.3. Related Research on Structural Holes

Structural holes refer to the voids between unconnected actors in a technological innovation network. If an actor connects two actors who are not directly connected, the actor is considered to occupy structural holes [38]. Structural holes depict the nonredundant connection between the two actors. Shi et al. [39] believed that firms occupying structural holes can quickly acquire nonredundant knowledge, accelerate the accumulation of coding knowledge, and deepen the understanding of existing knowledge. Long and Xinghua [40] believed that the widespread structural holes in the network are conducive to forming weak relationships between firms and encouraging firms to search for knowledge from a more extensive scope. However, some scholars believe that firms occupying structure holes do not establish a direct close connection with other connected firms, which will reduce the partner’s trust, aggravating the relationship breakdown between firms and, thus, hinder firms from acquiring knowledge from the outside [41].

Based on the above analysis, this study believes that structural holes, as a structural factor, have advantages in the network that can affect the depth and breadth of a knowledge search. More importantly, when structural holes directly or indirectly act on the level of interfirm relationships, they will also affect the depth and breadth of a knowledge search. Therefore, it is necessary to explore the moderating effect of structural holes further.

## 3. Research Hypotheses

### 3.1. The Impact of Divisive Faultlines on the Depth of Knowledge Search

In the technological innovation network, cooperation between firms often relies on the norms and conventions formed by historical cooperation experiences. Partners with solid relationships maintain intense and frequent contacts, forming cohesive subgroups, while partners with weak relationships are queued outside the cohesive subgroups [33]. A cohesive subgroup has the characteristics of a close and substantial connection and knowledge focus, which is conducive to firms’ in-depth searches. First, under the influence of inter-organization conventions, firms may not be willing to spend time and cost to establish new relationships but prefer to establish a deeper connection with historical partners, deepening the understanding of the existing domain knowledge, and to improve the efficiency of the knowledge reorganization and utilization in the process of innovation [42]. Second, under the effect of solid relationships, the close ties between firms establish a bridge for the flow of knowledge in the innovation network. The network members can search for knowledge quickly and accurately, reducing knowledge search costs [43]. Finally, as time goes by, the stronger the innovation network is, the higher the cohesion within the subgroup will be. Due to the existence of a high degree of trust mechanisms, members in the same subgroup are more willing to carry out knowledge reciprocity with local members, so that the knowledge can be focused on a specific field. The ability of members to absorb knowledge is enhanced, thus improving the depth of the knowledge search [44,45].

Take the filed patents of biologics-based drugs from the new York-based Pfizer in 2017 and 2018, for example. A strong, cohesive subgroup, the formation of divisive faultlines, could be observed between Pfizer and its collaboration partners. Among the 16 patents filed in 2017, 13 came either from the old partners (four in total) such as Merck, ABBVIE STEMCENTRX LLC, and BRISTOL-MYERS SQUIBB CO or from itself (nine in total). Similar cooperation subgroups could be found in the filed patents in 2018, with nine patents out of 10 coming from subgroups (five in total) or itself (four in total). A closer look into the knowledge of filed patents in these two years revealed a deepened depth of the knowledge search. Twenty-five out of 26 patents focused on the development of antibody or antibody–drug conjugates, which could be used to treat cancer. Among them, six patents, in collaboration with Merck, disclosed antibody therapies targeting a specific target, PD-L1. These patents described deep knowledge regarding the clinical treatments, ranging from antibody formulation to diagnostic antibody, as well as combinational treatment, of an anti-PD-L1 antibody and a target specific inhibitor. Based on the above analysis, we proposed the following hypothesis:

**Hypothesis** **1.** *Divisive faultlines have a positive impact on the depth of a knowledge search in the technological innovation network*.

### 3.2. The Impact of Divisive Faultlines on the Breadth of Knowledge Search

Divisive faultlines promote the depth of a knowledge search by forming highly cohesive subgroups, but the impact on the breadth of the knowledge search has two sides. On the one hand, when divisive faultlines are low, the network relationships are evenly distributed, and firms are either unfamiliar or very familiar, which is not conducive to the breadth of a knowledge search. Specifically, when firms are not familiar with each other, due to a lack of trust, the knowledge exchange between members of the subgroups is not deep, and the willingness to share knowledge is low, which hinders members in subgroups from searching for knowledge from the outside [18]. However, when firms are very familiar with each other, due to the cognitive constraints of homogenized knowledge, members of the subgroups rely excessively on the inherent innovation mode, making the subgroup internal information redundant and difficult to search for diversified knowledge [46]. On the other hand, with the strengthening of divisive faultlines, the distribution of relationships in the network become uneven. The differences in the degree of experience-sharing between members becomes more prominent, which quickly causes close connections between members of the subgroups and sparse connections between the subgroups. Halevy et al. [20] believed that the combination of close internal connections and appropriate bridging relationships can effectively increase the brokerage value and help network members search for diverse knowledge. The close contact within the subgroup provides members with stable resources and channels for information communication, reducing the knowledge search risk. The bridging relationship outside the subgroup establishes a communication channel for different subgroups, bringing heterogeneous knowledge to the subgroup members [47]. Especially, firms occupying an important position in the network can easily attract members outside the group to actively introduce their innovative resources and diversified knowledge to bridge the relationships built between groups [48]. In other words, strong relationships within subgroups and bridging relationships between subgroups increase the interactions between subgroups, and members can continuously search for new knowledge elements from the outside, expand the existing knowledge bases, and promote technological innovation in biopharmaceutical firms.

However, excessively high divisive faultlines have aggravated the factional gatherings among members, caused faction disputes between subgroups, and hindered general social exchanges among members in the technological innovation network. First, the interactions between members are limited to the group, and other resources such as knowledge and information are difficult to flow freely between the subgroups. As a result, the subgroup members can only obtain partial knowledge but cannot search for knowledge from a prominent scope [36]. Second, the enhancement of divisive faultlines will aggravate the degree of polarization of the subgroups in the technological innovation network, leading to limited cohesion between the subgroups. Further, amplifying the conflicts between members within and outside the subgroups, causing members inside and outside the subgroup to protect their knowledge, members of the network cannot obtain diverse knowledge. Third, the higher the divisive faultlines in the technological innovation network, the more significant the heterogeneity of knowledge between the subgroups, and the lower the transfer rate of knowledge between the subgroups, which will increase the difficulty of a knowledge search and is not conducive to the breadth of a knowledge search [37].

The “lightspeed” success of the Pfizer-BioNTech COVID-19 vaccine could be used to explain the impact of divisive faultlines and breadth of knowledge in the biopharmaceutical industry. An analysis of the collaboration partners of both companies before 2018 exhibited a rather confined innovation network. Besides, filed patents showed that Pfizer focused on the development of antibody treatments for cancer and BioNTech concentrated on RNA biotechnology, indicating a relatively narrow knowledge search within two companies resulting from a high level of divisive faultlines. However, the incentive to develop an effective influenza vaccine in 2018 drove Pfizer to find new collaborators and broaden the breadth of the knowledge search for vaccines outside its innovation networks connecting with old partners. Even though the first collaboration between Pfizer and BioNTech did not yield promising vaccines for the flu, an elementary subgroup free of conflicts and competition was formed between them, with BioNTech responsible for the vaccine discovery and Pfizer for the clinical trials and vaccine manufacturing. This kind of medium divisive faultline accelerated the development and wide distribution of the COVID-19 vaccine. Based on the above analysis, we proposed the following hypothesis:

**Hypothesis** **2.** *Divisive faultlines have an inverted U-shaped effect on the breadth of a knowledge search in the technological innovation network*.

### 3.3. The Moderating Effect of Structural Holes

Structural holes occupying the technological innovation network can strengthen the relationship between divisive faultlines and depth of a knowledge search. First, the occupants of structural holes can accurately and timely obtain a high innovation value from numerous knowledge streams and effectively control the direct contact between partners, saving maintenance partners. The time and effort spent on redundant relationships reduces the cost of a knowledge search [49]. Second, structural holes have a higher visibility in the network, and the degree of integration of the resources in the network is also higher [32]. Firms occupying higher structural holes are more likely to gain recognition from the subgroup members. Such recognition enables firms to identify with existing knowledge, which is conducive to firms focusing on refining and reorganizing the existing knowledge elements in innovation, improving their ability to absorb knowledge more. It has a positive effect on the depth of the knowledge search [39,44]. Finally, firms occupying structural holes have a higher impact on group activities. If members of the group are already familiar with this knowledge, they will be able to use more knowledge through reorganization to achieve innovation [50]. Therefore, the higher the divisive faultlines, the easier the firms occupying the structural holes may influence the members in the subgroups and spread the innovative knowledge, conducive to the in-depth mining and utilization of their knowledge.

However, the diverse knowledge brought by divisive faultlines has two sides for firms occupying higher structural holes. On the one hand, structural holes negatively regulate the relationship between divisive faultlines and the breadth of knowledge search. When a divisive faultline’s strength is low, high structural holes will make the network’s diversified structural characteristics more obvious. The members of the network will face the risk of information overload. They will easily fall into cognitive inertia, which weakens the firm’s willingness to acquire new knowledge [40]. Simultaneously, high structural holes reduce the trust between members, increase the cooperation costs and potential risks between firms, and make it more difficult for firms to obtain external resources, making enterprises more inclined to dig and use existing knowledge [41]. On the other hand, structural holes can break through the boundaries between subgroups, act as boundary crossers, and slow down the negative relationship between divisive faultlines and the breadth of the knowledge search. First, with the advantages of cross-firm boundaries and technical fields, firms occupying structural holes can cooperate with partners in other subgroups, conducive to forming bridging relationships between network subgroups to a certain extent, alleviating strong divisive faultlines. The structural isolation between groups reduces the difficulty of strong divisive faultlines for the knowledge flow and knowledge sharing between subgroups [51]. Second, structural holes establish a cooperative relationship between members inside and outside the subgroup, so that disconnected members have a common third party to monitor its partner behavior, curb opportunistic behavior, and prevent further destructive divisions [20]. Finally, with the help of the information advantages of structural holes, the occupants of structural holes can gain greater power in acting as an “information bridge”, which is conducive to establishing extensive cooperative relations between firms and other partners. Diversified knowledge, information, and other resources can be smoothly circulated in the network, prompting firms to search for diversified knowledge [49]. Based on the above analysis, we proposed the following hypotheses:

**Hypothesis** **3.** *Structural holes positively moderate the relationship between divisive faultlines and depth of the knowledge search. As the number of structural holes occupied by firms increases, divisive faultline’s positive effects on the depth of a knowledge search becomes stronger*.

**Hypothesis** **4.** *Structural holes negatively moderate the inverted U-shaped relationship between divisive faultlines and breadth of a knowledge search, making the inverted U-shaped curve flat: the positive relationship between divisive faultlines and breadth of a knowledge search becomes weaker, and the negative relationship between them also weakens*.

**Figure 1:**
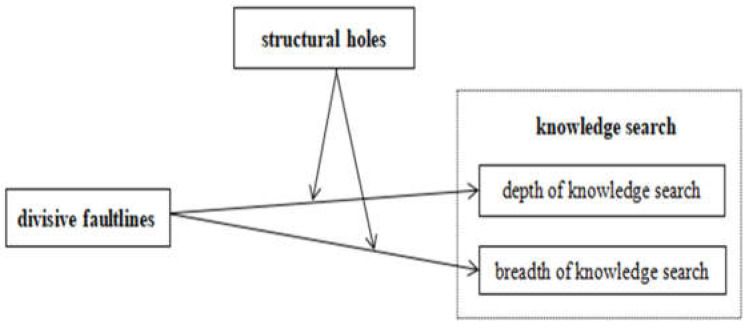
The theoretical model.

In summary, based on theoretical analysis and research hypotheses, the theoretical model constructed in this paper is shown in Figure 1.

## 4. Research Design

### 4.1. Data Source and Sample

As a typical knowledge-intensive industry, the biopharmaceutical industry integrates knowledge in different fields, such as biology, pharmacy, medicine, and biochemistry. Different from the traditional pharmaceutical industry, which mainly develops synthetic small molecular drugs, the biopharmaceutical industry concentrates on the development of biologics-based drugs, including peptides, antibody–drug conjugates, enzymes, and nucleic acid-based compounds. Compared with traditional drugs, biologics-based drugs outcompete them, owing to the advantages of high specificity and obtained immunogenicity, which have made the biopharmaceutical industry stand out in recent years. Especially after the outbreak of COVID-19 in 2020, many countries have successively listed COVID-19 as a strategic industry, which has promoted the development of the biopharmaceutical industry. Therefore, we used global biopharmaceutical firms as the research object.

A patent is the primary carrier of cooperation and innovation among firms. There are a large number of structured data fields in patent text, such as the patent number, patentee name, patentee code, application date, international patent classification (IPC), etc. The patentee and the patent right code provide data support for the construction of cooperation networks between firms, and the IPC classification number offers a feasible solution for the measurement of a knowledge search. Take the patent US2018296691-A1 that Pfizer and AbbVie jointly applied in 2017 as an example; the two firms have their own unique codes in the patents, namely PFIZ-C and ABBI-C, and there are three IPC classes in the patent, including A61K-031/704, A61K-047/68, and C07K-016/30. When measuring a knowledge search, we used the first four digits of the IPC (such as A61K or C07K). Therefore, this paper used patent data in the biopharmaceutical firms to verify the hypothesis. The process of collecting and cleaning patent data in biopharmaceutical firms includes the following steps:

First, the Derwent Innovations Index database contains the patent information of 41 patent institutions around the world, since 1963, covering more than 100 countries, which is the authoritative data source for global scientific and technological intelligence agencies. Therefore, we searched for biopharmaceutical patents from January 1, 2003 to December 31, 2018 through the Derwent Patent Database. Second, we cleaned the downloaded patent data. Since the patentee had many inconsistencies in the same firm’s names, such as name changes, abbreviated names, parent and subsidiary companies, and missing letters, this paper used the patentee code in the Derwent patent database to unify the names of the patentees. Simultaneously, we selected two or more patents with the patentee code, because the patents contained two or more different patent rights codes to represent the achievements of innovations between firms, so the code of the patentee was removed as the cooperative patent of the individual. In view of the fact that some firms’ temporary entry or exits will have a particular impact on the technological innovation network, this paper only retains firms that have participated in at least three collaborations in different years. Finally, we referred to previous studies [52], based on the cooperation between firms to apply for patents, we established a five-year mobile time window to divide the data from 2003 to 2018 into 11 time windows (i.e., 2003–2007, 2004–2008, … 2013–2017) and the corresponding technological innovation networks. Restricted by the lack of variable information and other problems, finally, we obtain the patent data of 509 biopharmaceutical firms. Therefore, this paper’s empirical analysis was based on the unbalanced panel data of 509 biopharmaceutical firms from 2003 to 2018 (N=1798).

### 4.2. Variable Definitions

#### 4.2.1. Dependent Variable

To more comprehensively measure the degree and scope of the knowledge search in a firm’s technological innovation network, we drew on the related research of Katila and Ahuja [28] to divide the knowledge search into two dimensions: depth of the knowledge search and breadth of the knowledge search. Depth of the knowledge search represents the degree of focus or specialization of knowledge, and breadth of the knowledge search represents the range or diversification of knowledge. Based on the availability of the existing research and patent data, this paper measured the depth of the knowledge search by calculating the average number of times that companies reused the knowledge in patent applications in year t. At the same time, by referring to the relevant research of scholars such as Rongkang et al. [53], the breadth of the knowledge search was measured by calculating the number of IPC categories (that is, taking the first four digits of the IPC patent number) contained in the patents filed by the firm in year t.

#### 4.2.2. Independent Variable

In this paper, based on the relevant research of Heidl et al. [17], we measured divisive faultlines mainly by the discreteness of the strength of the binary relation of the self-centered network. First, the historical relationship strength was measured by the duration of the relationship in years. According to the 5-year time window, the duration of the relationship between each self-centered network member pair in the past five years (t-5 to t-1) was calculated, with a value range of 0–5. For example, the historical relationship strength value was 1 if the member pair lasted for 1 year in the past 5 years. The strength value was 2 if the pair lasted for 2 years, and so on. Second, when we measured the uneven distribution of relationships among firms, it was measured by the standard deviation of the strength of the relationship between each pair of members in the self-centered network within a 5-year time window, because the standard deviation meant the discrete type of binary relationship distribution. The standard deviation was 0, which indicated that the relationship strength distribution between the member pairs in the self-centered network was uniform, which meant that the network’s divisive faultlines were low. The larger the standard deviation, it indicated that the distribution of the relationship strength between the pair of self-centered network members was exceptionally uneven, which meant that the network’s divisive faultlines were high.

#### 4.2.3. Moderator Variable

In this paper, based on the research of Jia and Yue [49] and Zhang and Luo [54], we used the constraint index in the Burt [38] indicator to calculate the firm’s structural holes in the technological innovation network from t-5 to t-1s year. Its calculation formula is as follows:

(1) $${\mathrm{Structural}\;\mathrm{Holes}}_{i}={\left(p_{\mathrm{ij}}+\underset{q}{\sum }p_{\mathrm{iq}}p_{\mathrm{qj}}\right)}^{2},q\neq i,j$$

where $p_{iq}$ denotes the investment proportion of firm i in the relationship of firm j in the technological innovation network, and $\sum_{q}p_{iq}p_{qj}$ is the total amount of indirect relationship of firm i in the network.

#### 4.2.4. Control Variables

Based on the research on the divisive faultlines and knowledge search in the technological innovation network, this paper argued that the factors at the technological innovation network level and the firm level will have an impact on the knowledge search, including network size, network density, betweenness centrality, knowledge base, and technological R&D capability. Network size: the larger the network’s size, the larger the number of firms in the technology innovation network, and the more complex the interaction process between members will be. Therefore, this paper controlled the network size by calculating the number of members directly related to the firm from t-5 to t-1 years. Network density: the greater the density of the technological innovation network, the closer the connection between members, and the greater the impact on the behavior of the firms in the network. The calculation formula is as follows: Network density=2L/(N ×(N−1)), where L is the actual relationship number in the technological innovation network, and N is the number of firms in the technological innovation network. This formula expresses the ratio of the actual relationship number to all possible relationship numbers in the five years from t-5 to t-1 in the technological innovation network. Betweenness centrality: the higher the centrality of a firm in the technological innovation network, the more important its position in the network, and the stronger the control over the surrounding firms. The calculation formula is as follows: $\mathrm{Betweenness}\;\mathrm{Centrality}=\sum_{b<c}\frac{m_{bc}\left(n_{a}\right)}{m_{bc}}$, where $m_{\mathrm{bc}}\left(n_{a}\right)$ represents the number of shortest paths for the two nodes of firm b and firm c through firm a in the technological innovation network, ${\mathrm{and}\;m}_{\mathrm{bc}}$ represents the number of shortest paths between the two nodes of firm b and firm c in the technology innovation network. Knowledge base: the larger the knowledge base is, the richer the firm’s knowledge elements, and the easier it is to search for knowledge in the technological innovation network. Therefore, this paper calculated the types of patent applications by firms in the five years from t-5 to t-1 [55]. Technology research and development capability: the stronger the technology research and development capabilities, the easier it is for firms to create new knowledge, affecting the knowledge search. Therefore, this paper calculated the total amount of patents accumulated by the firm over five years, from t-5 to t-1 [49].

### 4.3. Empirical Model

This research divided the knowledge search into two dimensions: the depth of a knowledge search (KSD) and the breadth of a knowledge search (KSB). For Hypothesis (H1), KSD is the dependent variable, and divisive faultlines (DF) are the independent variable, with network size (NS), network density (ND), betweenness centrality (BC), knowledge base (KB), and technology research and development capability (TRDC) being the control variables. Taking into account the lag between the independent variable and the dependent variable, we used the dependent variable of year t (such as 2008) to perform regression calculations on the independent variable of year t-5~t-1 (such as 2003~2007), and the measurement model is as follows:

(2) $${\mathrm{KSD}}_{i,t}=\alpha +\beta_{1}{\mathrm{DF}}_{i,t-5\sim t-1}+\beta_{i}{\mathrm{controls}}_{i,t-5\sim t-1}+\varepsilon_{i,t}$$

For Hypothesis (H2), KSB is the dependent variable, and DF is the independent variable, with NS, ND, BC, KB, and TRDC being the control variables. Similarly, the measurement model designed by us is as follows:

(3) $${\mathrm{KSB}}_{i,t}=\alpha +\beta_{1}{\mathrm{DF}}_{i,t-5\sim t-1}+\beta_{2}{\mathrm{DF}}_{i,t-5\sim t-1}^{2}+\beta_{i}{\mathrm{controls}}_{i,t-5\sim t-1}+\varepsilon_{i,t}$$

For Hypothesis (H3), KSD is the dependent variable, and DF is the independent variable; structural holes (SH) are the moderator variable, with NS, ND, BC, KB, and TRDC being the control variables. The model is as follows:

(4) $$KSD_{i,t}=\alpha +\beta_{1}DF_{i,t-5\sim t-1}+\beta_{2}SH_{i,t-5\sim t-1}+\beta_{3}DF_{i,t-5\sim t-1}\times \beta_{4}SH_{i,t-5\sim t-1}\;+\beta_{i}controls_{i,t-5\sim t-1}+\varepsilon_{i,t}$$

For Hypothesis (H4), KSB is the dependent variable, and DF is the independent variable; SH is the moderator variable, with NS, ND, BC, KB, and TRDC being the control variables. Similarly, the model is as follows:

(5) $$KSB_{i,t}=\alpha +\beta_{1}DF_{i,t-5\sim t-1}+\beta_{2}DF_{i,t-5\sim t-1}^{2}+\beta_{3}SH_{i,t-5\sim t-1}\;\;+\beta_{4}DF_{i,t-5\sim t-1}\times \beta_{5}SH_{i,t-5\sim t-1}\;\;+\beta_{6}DF_{i,t-5\sim t-1}^{2}\times \beta_{7}SH_{i,t-5\sim t-1}+\beta_{i}controls_{i,t-5\sim t-1}+\varepsilon_{i,t}$$

where i represents the firm, t represents the time, α represents the constant term, $\beta_{i}$ is the coefficient, and ε is the coefficient.

## 5. Empirical Results

### 5.1. Descriptive Statistics and Relevance Analysis

Table 1 shows the results the of descriptive statistics and correlation analysis of the variables. It can be seen from Table 1 that the mean value of the depth of the knowledge search was 0.508, and the standard deviation was 0.198, while the mean value of the breadth of the knowledge search was 4.862, and the standard deviation was 2.184.

This showed little differences in the mining and utilization of existing knowledge resources by firms. Still, there is a big difference in obtaining diversified knowledge among different firms. The average value of the divisive faultlines was 0.904, which indicates that the distribution of relationships among firms in the technological innovation network is uneven, affecting the adoption of different knowledge search strategies by firms. At the same time, as can be seen from Table 1, the influential coefficients of divisive faultlines on the depth of the knowledge search and the breadth of the knowledge search were 0.051 ** and 0.040 *, respectively, indicating that there may be a significant positive correlation between the divisive faultlines and the depth of the knowledge search, as well as between the divisive faultlines and the breadth of the knowledge search. The influence coefficients of the structural holes on the depth of the knowledge search and the breadth of the knowledge search were 0.046 * and −0.344 ***, respectively, indicating that there was a significant positive correlation between the structural holes and the depth of the knowledge search, while there was a significant negative correlation between the structural holes and the breadth of the knowledge search. However, a Pearson correlation analysis did not consider the influence of the control variables, which requires further analysis and testing.

### 5.2. Multicollinearity Test

It can be seen from Table 1 that the correlation coefficient among some variables was significant. To test whether there was a multicollinearity among variables, the variance inflation factor and tolerance of all the variables were calculated in this paper, and the results are shown in Table 2. The results showed that the highest variance inflation factor value of all the variables was 3.40, which was lower than the recommended upper limit of 10. The minimum tolerance value was 0.294, which was greater than the recommended lower limit of 0.100, indicating no obvious multicollinearity problem among the variables.

### 5.3. Regression Analysis

To overcome the problem of outliers in the data, before the regression analysis, we performed a logarithmic transformation on some variables with a larger value range, such as the breadth of the knowledge search, divisive faultlines, network size, betweenness centrality, etc. and increased the value of the variable involved in the logarithmic conversion by 1 if the logarithm could not be taken. At the same time, to avoid the problem of multicollinearity caused by the interaction term between the independent variable and the moderator variable, we centralized the independent variable and the moderator before constructing the interaction term. With the help of Stata 15.0 software, a hierarchical regression model was used to verify all the hypotheses.

#### 5.3.1. The Impact of Divisive Faultlines on Knowledge Search in Technological Innovation Network

This part empirically examined the impact of divisive faultlines on the depth of the knowledge search and the breadth of the knowledge search. We used a hierarchical regression model for the analysis. The regression results are shown in Table 3.

Model 1 was the basic model to test the influence of the control variables on the dependent variables. Based on Model 1, an independent variable was added to construct Model 2 to verify the relationship between the divisive faultlines and the depth of the knowledge search. The results showed a significant positive relationship between the divisive faultlines and the depth of the knowledge search (β = 0.798, *p* < 0.01). Therefore, Hypotheses 1 was verified. It can be seen that, as the divisive faultlines increased, the cohesion of the subgroups became higher. Under the effect of interfirm practices, lower knowledge search costs, and robust absorptive capacity, members can easily keep close contact with each other, enhance the understanding of each other’s knowledge, and improve the depth of knowledge search.

Model 4 was the basic model to test the influence of the control variable on the dependent variable. Based on Model 4, the first quadratic of the independent variables was added to construct Model 5 to verify the relationship between the divisive faultlines and the breadth of the knowledge search. The results showed that the regression coefficient of the influence of the square term of divisive faultlines on the breadth of the knowledge search was significantly negative (β = −1.633, *p* < 0.01), and the regression coefficient of the influence of the primary term of the divisive faultlines on the breadth of the knowledge search was significantly positive (β = 1.971, *p* < 0.01). Therefore, Hypothesis 2 was verified. It can be seen that the enhancement of divisive faultlines will help firms obtain more novel knowledge from the outside, thereby increasing the breadth of the knowledge search. However, when the divisive faultlines exceeded a particular critical value, the structural isolation of the subgroups became more obvious, and the factional disputes between the subgroups intensified, hindering the knowledge flow between the subgroups and thereby reducing the breadth of the knowledge search.

#### 5.3.2. The Moderating Effect of Structural Holes

In this part, we investigate the moderating effect of the structural holes on the divisive faultlines and the depth of the knowledge search, and the moderating effect of structural holes on the divisive faultlines and the breadth of the knowledge search. The regression results are shown in Table 3.

**Figure 2:**
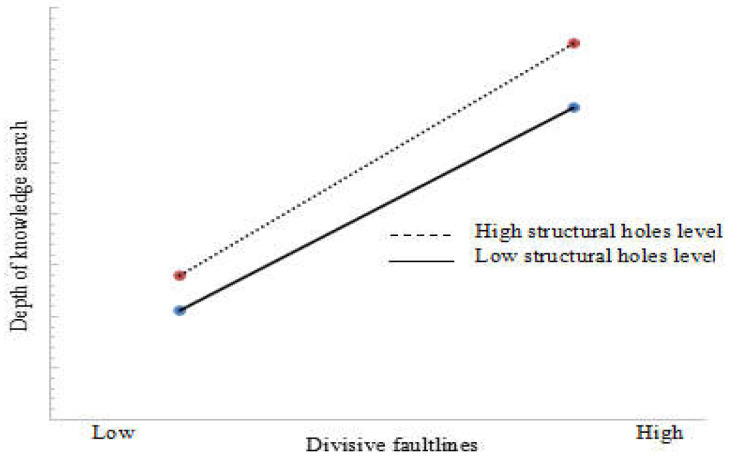
The moderating effect of structural holes on divisive faultlines and the depth of the knowledge search.

Based on Model 2, interaction terms of the moderating variables and independent variables were added to construct Model 3 to verify the structural holes’ moderating effect on the divisive faultlines and the depth of the knowledge search. The results showed that the interaction terms of the structural holes and the divisive faultlines significantly impacted the depth of the knowledge search (β = 0.242, *p* < 0.01). Therefore, Hypothesis 3 was verified. To more clearly understand the moderating effect of the structural holes between divisive faultlines and the depth of the knowledge search, we respectively selected the mean value of the independent variables and adjusting variables, plus or minus one standard deviation, and substituted them into the regression model to make a plot, as shown in Figure 2. When the level of the structural holes was high, a divisive faultline’s impact on the depth of the knowledge search was significantly greater than when the level of the structural holes was low. It can be seen that when a firm occupies high structural holes, the advantage it has in technological innovation networks can strengthen the relationship between divisive faultlines and the depth of the knowledge search to a certain extent.

**Figure 3:**
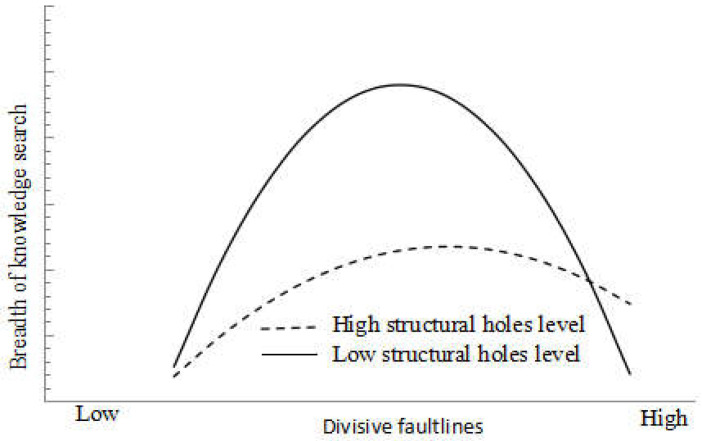
The moderating effect of structural holes on the divisive faultlines and the breadth of the knowledge search.

Based on Model 5, interaction terms of moderating variables and independent variables were added to construct Model 6 to verify the structural holes’ moderating effects on the divisive faultlines and the breadth of the knowledge search. The results showed that the regression coefficient of the interaction term of the divisive faultlines square term and structural holes on the breadth of the knowledge search were significantly positive (β = 3.369, *p* < 0.05), and the regression coefficient of the primary term of the divisive faultlines on the breadth of the knowledge search was significantly negative (β = −4.330, *p* < 0.05). Therefore, Hypothesis 4 was verified. To more clearly understand the moderating effects of the structural holes between the divisive faultlines and the breadth of the knowledge search, in the same way, we drew a diagram of the moderating effects of the structural holes on the divisive faultlines and the breadth of the knowledge search, as shown in Figure 3. When the structural holes’ level was higher, the positive relationship between the divisive faultlines and the breadth of the knowledge search became weaker. The negative relationship between the divisive faultlines and the breadth of the knowledge search also became vulnerable, making the entire inverted U-shaped curve flat. It can be seen that, on the one hand, high structural holes not only reduced the trust between firms, increasing the probability of opportunism and free-riding behavior, but also weakened the willingness of firms to acquire new knowledge, thereby reducing the positive impact of divisive faultlines on the breadth of the knowledge search. On the other hand, the advantages of high structural holes in technological innovation networks can reduce the difficulty of strong divisive faultlines on the knowledge flow and knowledge sharing between the subgroups, thereby reducing the negative impact of divisive faultlines on the breadth of the knowledge search.

### 5.4. Robustness Test

In order to verify the robustness of the experimental results, this paper used the efficiency index of structural holes to replace the constraint index of structural holes [37] to eliminate the influence of the single-variable measurement method on the regression results. The regression results are shown in Table 4.

The results showed a positive relationship between the divisive faultlines and the depth of the knowledge search (β = 1.377, *p* < 0.01); the results still supported Hypothesis 1. The regression coefficient of the influence of the square term of the divisive faultlines on the breadth of the knowledge search was significantly negative (β = −0.929, *p* < 0.01), and the regression coefficient of the influence of the primary term of the divisive faultlines on the breadth of the knowledge search was significantly positive (β = 1.102, *p* < 0.01); the results still supported Hypothesis 2. The interaction terms between the structural holes and divisive faultlines had a significant positive impact on the depth of the knowledge search (β = 1.063, *p* < 0.01); Hypothesis 3 was still supported. The regression coefficient of the interaction term between the square term of the divisive faultlines and structural holes on the breadth of the knowledge search was significantly positive (β = 1.780, *p* < 0.05), and the regression coefficient of the interaction term between the primary term of the divisive faultlines and the structural holes on the breadth of the knowledge search was significant and negative (β = −2.626, *p* < 0.05); Hypothesis 4 was still supported. Therefore, the hypotheses test results in this paper were robust.

## 6. Conclusions

Based on 509 global biopharmaceutical firms from 2003 to 2018, this paper explored the impact of divisive faultlines in technology innovation networks on the depth of the knowledge search and the breadth of the knowledge search from the meso subgroup level and the moderating effect of SH on them. The main conclusions of this study were as follows:

(1) Divisive faultlines positively impacted the depth of the knowledge search in the technological innovation network. By improving the cohesion of the subgroups of the technological innovation network, divisive faultlines can promote frequent cooperation and knowledge-sharing among members of the subgroup, thus enhancing the depth of the knowledge search. This research conclusion was in line with the views of Xinhua et al. [33] and Reagans et al. [56]. (2) Divisive faultlines have an inverted U-shaped effect on the breadth of the knowledge search in the technological innovation network. With the increase of the divisive faultlines, the breadth of the knowledge search showed an increasing trend. However, when the divisive faultlines exceeded a certain critical value, the breadth of the knowledge search tended to decrease. This indicates that moderate divisive faultlines are the most favorable for the breadth of a knowledge search. The highly divisive faultlines reduced the cohesion between the subgroups and increased the conflict between the internal and external subgroups. To a certain extent, this research conclusion supported the views of Heidl et al. [17]. (3) Structural holes played a positive moderating role in the positive relationship between the divisive faultlines and the depth of the knowledge search. Firms occupying structural holes can not only effectively control the relationship between partners and strengthen the cooperation between members of the subgroup under the control advantage but, also, reduce the cost and risk of the knowledge search, making the members of the network more willing to cooperate frequently with the occupiers of the structural holes, thereby deepening the depth of the knowledge search. (4) Structural holes negatively moderate the inverted U-shaped relationship between the divisive faultlines and the breadth of the knowledge search, making the inverted U-shaped curve flat. It can be seen that, when divisive faultlines between firms are low, the high-structure holes will have a negative moderating effect on the positive relationship between the divisive faultlines and the breadth of the knowledge search. However, when the divisive faultlines between firms are strong, structural holes can break through the boundaries between subgroups and act as boundary crossers, which reduces the structural isolation between subgroups by strong divisive faultlines and the difficulty of the knowledge flow between the subgroups, thereby weakening the negative influence of divisive faultlines on the breadth of the knowledge search.

This study obtained the following policy suggestions through empirical research. First, the development process of new biologics-based drugs is time-consuming and requires significant investment, similar to the development of small-molecule drugs; a new biologics-based drug project also followed several stages before receiving the FDA approval to be launched on the market. The project started with target identification, biological mechanism elucidation, and lead compound designs before the testing on animal models in the preclinical trial. After the success in the preclinical experiments, the drug will be examined in clinical phase I to III and, finally, prepared for new drug applications. Therefore, biopharmaceutical firms need rich knowledge and advanced technology in the R&D process; cooperation with other research and development agencies is of great importance. Take the ALK-1 monoclonal antibody for liver cancer treatment, for example. The drug was developed by Pfizer in the early stage and two phase I clinical trials and verifications were completed in 2014. Later, Pfizer sought collaboration with Kintor Pharma-B to carry out global multicenter phase II clinical trials with the joint dosing of different drugs, promoting the successful development and manufacturing of the drug that was the first approved monoclonal antibody drug worldwide in 2018.

Second, in the process of cooperation between enterprises, there is also competition, which makes the strength of the relationship between enterprises different. Divisive faultlines caused by the uneven distribution of the strength of the relationships between firms could greatly affect the innovation process in the biopharmaceutical industry. Divisive faultlines enhance the cohesion between familiar firms, increasing the depth of knowledge search within the technology innovation network. The deepened depth of knowledge search required very frequent revisits of the existing knowledge, which accelerated the processes to identify promising targets, to figure out the biochemical mechanisms of the diseases, and to explore potential compounds for treatment. The moderate divisive faultlines are beneficial to affect the breadth of the knowledge search; the breadth of the knowledge search could broaden the horizons of the existing innovation network, introduce diversified knowledge from external sources and benefitting the sustainable development of the biopharmaceutical industry. Therefore, when selecting partners, firms should choose to cooperate with partners they are familiar with to ensure the smooth development of new drugs through in-depth knowledge. Simultaneously, biopharmaceuticals need to search for knowledge extensively in the research and development process. In order to reduce the negative impact of the divisive faultlines on the breadth of the knowledge search, when choosing partners, biopharmaceutical firms need to understand the relationship mode of the partners, and according to our own research and development, the ability to choose innovation partners, improving the R&D efficiency of biopharmaceuticals. Moreover, some biopharmaceutical firms occupying important positions can use their own advantages to drive the development of the entire biopharmaceutical industry, even if there are divisive faultlines in the technological innovation network. Therefore, firms occupying structural holes must make full use of their information advantages and control the advantages to seize the opportunity to promote the innovation performance of the firm. Especially small- and medium-sized biopharmaceutical firms, when they cooperate with other firms to build an innovation network, need to create and occupy structural holes to promote the sustainable development of the firm.

Finally, for researchers in both academia and the industry, a high level of divisive faultlines is necessary to make innovative breakthroughs in many diseases with well-defined biological mechanisms. Strong and long-term relationships are the prerequisite to obtaining promising treatments for diseases. Take cancer treatment research, for example. The causing mechanism of cancer is much better understood now than 20 years ago. Based on the explored knowledge of oncology, targeted therapy (such as antibody–drug conjugates) and immunotherapy were invented and showed greater benefits compared with chemotherapy or radiation therapy. The cooperation on developing antibody–drug conjugates between Celltech and Wyeth (acquired by Pfizer later) since 1991 has yielded two FDA-approved drugs, mylotarg and besponsa in 2017. In contrast, for diseases without elucidated working mechanisms like neurodegenerative diseases such as Parkinson’s diseases and Alzheimer’s diseases, a strong cohesive collaboration group usually restricts the breadth of knowledge researchers could explore, which would make them override other promising treatment methods and might result in failures of the research project. The ending of the project on treatments for Alzheimer’s disease and Parkinsonism from Pfizer in 2018 could result from the high-level divisive faultlines and lack of broad knowledge search. For company leaders making development policies, a strong collaboration with old partners (corporations or research institutes) should kept to evoke biotechnology innovation breakthroughs and promote drug marketing, which would nourish a healthy relationship in return. At the same time, a medium-level collaboration relationship could be established with “unfamiliar companies” with biotechnology outside the internal innovation networks. This kind of relationship could make good use of their advantages from both sides and avoid the conflicts of interest to a large extent, accelerating the marketing of new biologics-based drugs and broadening the research area for future development. The success of the Pfizer-BioNTech COVID-19 vaccine is a good example, with BioNTech providing mRNA biotechnology and Pfizer providing clinical testing platforms and manufacturing.

However, this study still has some expandable directions. This paper only considered the impact of divisive faultlines caused by the uneven distribution of the relationship strength between firms on the knowledge search, which has certain limitations. In the future, we can analyze the reasons for the formation of divisive faultlines and the impact on the knowledge search from the heterogeneity of the firm itself. At the same time, this paper conducted research based on patent data. Patent data generally measures the explicit knowledge of the firm but cannot measure the tacit knowledge. In the future, the firm’s tacit knowledge can be obtained through questionnaire surveys, yearbooks, and other methods. In addition, to understand the results of collaborative research between biopharmaceutical firms, the development of key drugs can be tracked through patents in the future.

## Figures and Tables

**Table 1:** Descriptive statistics and person correlation matrix.

Variable	Mean	Sd	1	2	3	4	5	6	7	8	9
1. KSD	0.508	0.198	1								
2. KSB	4.862	2.184	0.550 ***	1							
3. DF	0.904	0.313	0.051 **	0.040 *	1						
4. SH	0.483	0.193	0.046 *	−0.344 ***	0.395 ***	1					
5. NS	7.923	6.077	−0.032	0.347 ***	−0.146 ***	−0.644 ***	1				
6. ND	0.426	0.302	0.006	−0.256 ***	0.247 ***	0.633 ***	−0.283 ***	1			
7. BC	0.693	1.623	−0.096 ***	0.343 ***	−0.150 ***	−0.452 ***	0.527 ***	−0.318 ***	1		
8. KB	9.195	3.946	−0.239 ***	0.575 ***	0.028	−0.452 ***	0.448 ***	−0.322 ***	0.522 ***	1	
9. TRDC	12.043	14.269	−0.104 ***	0.563 ***	0.041 *	−0.469 ***	0.595 ***	−0.361 ***	0.551 ***	0.781 ***	1

Notes: *n* = 1798; ***, **, and * denote statistical significances at the 1%, 5%, and 10% levels, respectively.

**Table 2:** Multicollinearity test.

Variable Name	Variable Representation	Variance Inflation Factor	Tolerance
Divisive Faultlines	DF	1.32	0.760
Structural Holes	SH	3.28	0.305
Network Size	NS	2.48	0.403
Network Density	ND	1.85	0.541
Betweenness Centrality	BC	1.69	0.592
Knowledge Base	KB	2.81	0.356
Technology research and development Capability	TRDC	3.40	0.294

**Table 3:** Regression results.

Variable	KSD			KSB		
	Model 1	Model 2	Model 3	Model 4	Model 5	Model 6
NS	−0.013 *	0.002	0.002	0.016	0.019	0.018 *
	(0.007)	(0.004)	(0.004)	(0.015)	(0.015)	(0.011)
ND	0.290 ***	0.203 ***	0.165 ***	−0.029	0.002	0.249 ***
	(0.023)	(0.015)	(0.014)	(0.030)	(0.031)	(0.030)
BC	−0.098 ***	−0.049 ***	−0.035 ***	0.211 ***	0.202 ***	0.084 ***
	(0.013)	(0.007)	(0.006)	(0.037)	(0.036)	(0.027)
KB	−0.015	−0.003	−0.001	0.013	0.000	−0.031
	(0.016)	(0.009)	(0.009)	(0.040)	(0.040)	(0.030)
TRDC	0.016	−0.058 ***	−0.036 ***	0.242 ***	0.256 ***	0.087 ***
	(0.010)	(0.006)	(0.006)	(0.028)	(0.030)	(0.023)
DF		0.798 ***	0.706 ***		1.971 ***	2.057 ***
		(0.022)	(0.020)		(0.378)	(0.349)
DF2					−1.633 ***	−1.293 ***
					(0.281)	(0.277)
SH			0.241 ***			−1.791 ***
			(0.024)			(0.073)
DF × SH			0.242 ***			−4.330 **
			(0.069)			(1.779)
DF^2^ × SH						3.369 **
						(1.308)
_cons	0.499 ***	0.120 ***	0.010	1.353 ***	0.786 ***	1.852 ***
	(0.041)	(0.023)	(0.022)	(0.078)	(0.135)	(0.123)
N	1798.000	1798.000	1798.000	1798.000	1798.000	1798.000
R2	0.5585	0.8614	0.8769	0.4072	0.4253	0.6840
Wald chi2	502.79	3336.42	5573.28	289.57	366.70	1063.87

Notes: The statistics in parentheses are *t*-statistics. ***, **, and * denote the statistical significance at the 1%, 5%, and 10% levels, respectively.

**Table 4:** Results of the robustness test.

Variable	KSD			KSB		
	Model 1	Model 2	Model 3	Model 4	Model 5	Model 6
NS	−0.019	−0.000	−0.013 **	0.018 *	0.022 **	0.021 **
	(0.013)	(0.008)	(0.006)	(0.010)	(0.010)	(0.010)
ND	0.685 ***	0.432 ***	0.098 ***	0.030 *	0.062 ***	−0.041 *
	(0.035)	(0.024)	(0.019)	(0.015)	(0.017)	(0.025)
BC	−0.333 ***	−0.193 ***	−0.136 ***	0.153 ***	0.145 ***	0.153 ***
	(0.024)	(0.014)	(0.010)	(0.011)	(0.011)	(0.011)
KB	−0.031	0.001	−0.011	−0.002	−0.008	−0.008
	(0.023)	(0.014)	(0.010)	(0.016)	(0.016)	(0.016)
TRDC	0.068 ***	−0.083 ***	−0.045 ***	0.094 ***	0.105 ***	0.113 ***
	(0.013)	(0.009)	(0.006)	(0.010)	(0.010)	(0.010)
DF		1.377 ***	1.382 ***		1.102 ***	1.208 ***
		(0.029)	(0.019)		(0.148)	(0.167)
DF2					−0.929 ***	−1.027 ***
					(0.109)	(0.129)
SH			−1.392 ***			−0.425 ***
			(0.053)			(0.064)
DF × SH			1.063 ***			−2.626 **
			(0.135)			(1.065)
DF^2^ × SH						1.780 **
						(0.861)
_cons	−0.858 ***	−1.459 ***	−0.657 ***	0.412 ***	0.079	0.299 ***
	(0.055)	(0.030)	(0.042)	(0.029)	(0.054)	(0.065)
N	1798.000	1798.000	1798.000	1798.000	1798.000	1798.000
Pseudo R2	0.0285	0.0434	0.0465	0.0153	0.0160	0.0165
Log−likelihood	−1365.9876	−1345.0422	−1340.6433	−2405.076	−2403.1779	−2402.0112

Notes: The statistics in parentheses are *t*-statistics. ***, **, and * denote the statistical significance at the 1%, 5%, and 10% levels, respectively.

## Data Availability

All tables and figures in the paper are made by the authors. The data are taken from the Derwent Innovations Index database. The data in this paper can be obtained from the authors.
